# Parkinson's disease brain mitochondria have impaired respirasome assembly, age-related increases in distribution of oxidative damage to mtDNA and no differences in heteroplasmic mtDNA mutation abundance

**DOI:** 10.1186/1750-1326-4-37

**Published:** 2009-09-23

**Authors:** Charles R Arthur, Stephanie L Morton, Lisa D Dunham, Paula M Keeney, James P Bennett

**Affiliations:** 1 Morris K Udall Parkinson's Disease Research Center of Excellence, University of Virginia School of Medicine, Charlottesville, VA 22908, USA

## Abstract

**Background:**

Sporadic Parkinson's disease (sPD) is a nervous system-wide disease that presents with a bradykinetic movement disorder and is frequently complicated by depression and cognitive impairment. sPD likely has multiple interacting causes that include increased oxidative stress damage to mitochondrial components and reduced mitochondrial bioenergetic capacity. We analyzed mitochondria from postmortem sPD and CTL brains for evidence of oxidative damage to mitochondrial DNA (mtDNA), heteroplasmic mtDNA point mutations and levels of electron transport chain proteins. We sought to determine if sPD brains possess any mtDNA genotype-respiratory phenotype relationships.

**Results:**

Treatment of sPD brain mtDNA with the mitochondrial base-excision repair enzyme 8-oxyguanosine glycosylase-1 (hOGG1) inhibited, in an age-dependent manner, qPCR amplification of overlapping ~2 kbase products; amplification of CTL brain mtDNA showed moderate sensitivity to hOGG1 not dependent on donor age. hOGG1 mRNA expression was not different between sPD and CTL brains. Heteroplasmy analysis of brain mtDNA using Surveyor nuclease^® ^showed asymmetric distributions and levels of heteroplasmic mutations across mtDNA but no patterns that statistically distinguished sPD from CTL. sPD brain mitochondria displayed reductions of nine respirasome proteins (respiratory complexes I-V). Reduced levels of sPD brain mitochondrial complex II, III and V, but not complex I or IV proteins, correlated closely with rates of NADH-driven electron flow. mtDNA levels and PGC-1α expression did not differ between sPD and CTL brains.

**Conclusion:**

PD brain mitochondria have reduced mitochondrial respiratory protein levels in complexes I-V, implying a generalized defect in respirasome assembly. These deficiencies do not appear to arise from altered point mutational burden in mtDNA or reduction of nuclear signaling for mitochondrial biogenesis, implying downstream etiologies. The origin of age-related increases in distribution of oxidative mtDNA damage in sPD but not CTL brains is not clear, tracks with but does not determine the sPD phenotype, and may indicate a unique consequence of aging present in sPD that could contribute to mtDNA deletion generation in addition to mtDNA replication, transcription and sequencing errors. sPD frontal cortex experiences a generalized bioenergetic deficiency above and beyond aging that could contribute to mood disorders and cognitive impairments.

## Background

Parkinson's disease (PD) is the most commonly occurring neurodegenerative movement disorder of adults and occurs sporadically (sPD) in >90% of cases. sPD is now recognized to be a nervous system-wide disorder with a temporal progression of protein aggregation pathology that begins in gut enteric neurons, then enters vagal and olfactory neurons before appearing in midbrain substantia nigra [1-6]. Death of nigral dopaminergic neurons heralds the onset of the diagnostic bradykinetic movement disorder, while pathology continues to spread rostrally into limbic and eventually frontal cortex. Recent data are consistent with early functional cerebral cortical involvement that precedes cortical Lewy pathology [7]. Less intense neurodegeneration in locus coereuleus and midbrain raphe nuclei, combined with cortical Lewy neurite and Lewy body formation, likely contribute to the frequent comorbidities of depression and cognitive impairment seen in many sPD patients [8].

Currently five monogenic causes of parkinsonism are known that produce clinical phenotypes variably similar to, and in some cases virtually identical with sPD [9-11]. Mechanisms of pathogenesis in these autosomal genetic forms of parkinsonism appear to include disruptions of mitochondrial morphology and function [12,13]. In sPD a substantial body of work has demonstrated associations in brain and peripheral tissues with impaired mitochondrial bioenergetics and increased oxidative stress [14,15]. Although the causality of mitochondrial deficits for contributing to human sPD pathogenesis is not proven, a compelling rodent model of nigral dopaminergic degeneration and protein aggregation reminiscent of Lewy pathology is observed after chronic systemic treatment with rotenone, a mitochondrial electron transport chain poison that inhibits electron transfer at the ubiquinone reduction site in complex I [16,17]. Rotenone toxicity to mammalian cultured cells [18] or rat dopaminergic neurons [19] derives from increased oxidative stress and is eliminated by expression of a yeast complex I insensitive to rotenone, implying that rotenone neurodegeneration *in vivo *derives from oxidative stress generated at complex I. Thus, sPD and monogenic parkinsonisms appear to share disrupted mitochondrial function with altered bioenergetics as common characteristics that are potentially pathogenic.

Assessing mitochondrial bioenergetic function and consequences of bioenergetic inefficiency is limited in frozen postmortem sPD brain. Traditionally, maximal catalytic activities of individual ETC complexes have been assayed, yielding a frequent finding that complex I activity is reduced. However, these assays of isolated complex activities do not necessarily provide insight into respiratory capacity, coupling of respiration to ATP synthesis and rate of production of reactive oxygen species (ROS). Freezing disrupts mitochondrial capacity to generate proton gradients across the inner membrane, which are necessary for ATP synthesis and are inversely related to ROS production.

In our earlier work we used complex I immunocapture antibodies to show that sPD brain mitochondria had elevated oxidative damage to several complex I proteins, and that the levels of these oxidized complex I subunit proteins inversely correlated with NADH-driven electron flow [20]. These results suggested that the increased oxidative damage to complex I proteins in sPD brains were causally involved in slowing NADH-driven electron flux. We also showed that exposure of CTL mitochondria to high [NADH] produced oxidative damage to complex I proteins similar to what we found in sPD samples. This suggested that at least some of the complex I oxidative damage could be internally generated.

In the present study we have extended our analysis to measure in sPD brain mitochondria the levels per unit mitochondrial mass of multiple ETC proteins that are believed to functionally associate with each other in a respiratory unit known as the "respirasome" [21,22]. Because these ETC proteins from all five complexes were reduced in sPD brain mitochondria, we explored whether abnormalities in sPD mtDNA could account for this deficiency. We used a mismatch-cleaving endonuclease (Surveyor^®^) to search for low-abundance heteroplasmic mutations in multiple overlapping PCR fragments of sPD mtDNA [23,24]. Finally, we quantified the distribution of oxidative mtDNA lesions in sPD brain by assessing inhibition of qPCR amplification by treatment with the DNA glycosylase hOGG1, a component of the mitochondrial base excision repair pathway. We found that neither mtDNA heteroplasmic mutation abundances nor oxidative mtDNA damage provided insight into why sPD respirasome assembly appears defective. In addition, levels of reduced sPD brain mitochondrial respiratory proteins from complexes II, III and V correlated closely with rates of NADH-driven electron flow, implying a functional relationship to respirasome function.

## Results

### Postmortem brain samples

Clinical histories, cause of and age at death, sex, and postmortem interval between death and dissected sample placement into -80°C freezer are shown in Additional Files 1 for CTL and 2 for sPD. The average age at death for CTL samples was 67.0 ± 13.4 years and for sPD samples was 77.8 ± 4.9 years with no significant difference (p = 0.08). CTLs consisted of 7 males and 3 females (70% males) and the sPDs were composed of 5 males and 3 females (62.5% males) with no significant difference (p = 0.73). Postmortem interval for CTLs was 8.65 ± 4.77 hours and for sPDs 12.66 ± 4.15 hours (p = 0.11). (Additional File 3)

### ETC Complex I Subunits Are Significantly Reduced in sPD Frontal Cortex

**Figure 1:**
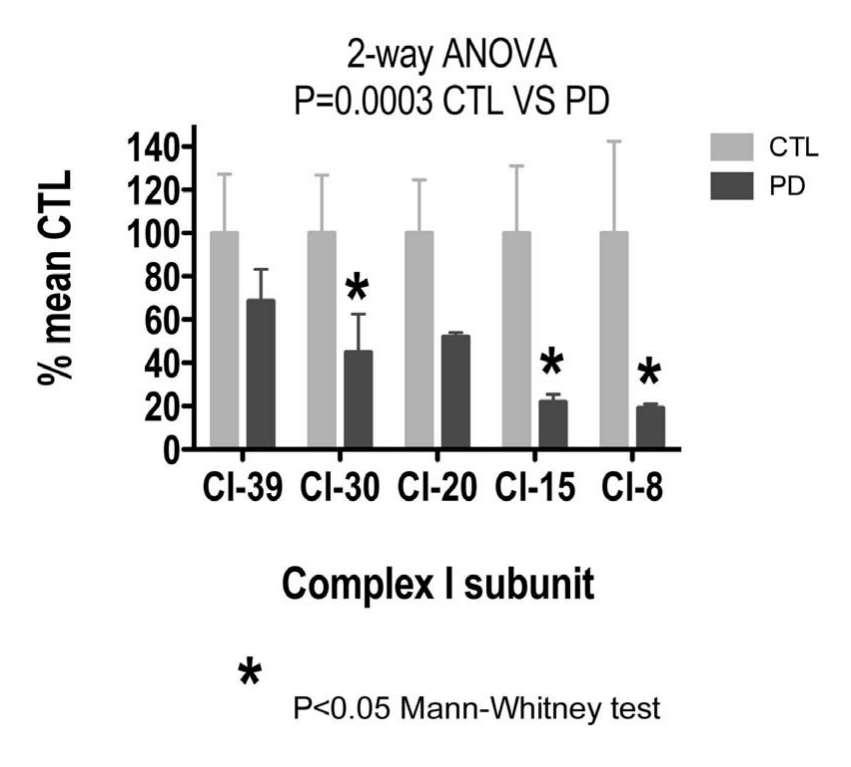
**Levels of Complex I subunit proteins in sPD brain mitochondria**. Individual protein band intensities were normalized to porin intensity in each sample and expressed in terms of mean CTL levels. A 2-way ANOVA across subunits and disease showed a significant interaction. Mann-Whitney test showed significant reductions for 30 kDa, 15 kDa and 8 kDa subunits in sPD samples.

Figure 1 shows the results of Western blot assays for ETC subunits of Complex I in sPD and CTL brain mitochondria. Band integrated intensities in each sample were normalized against mitochondrial porin, an abundant outer mitochondrial membrane protein, in each sample as a marker for mitochondrial mass. The porin-normalized CTL values were averaged, and each individual sPD or CTL subunit level was expressed as a percentage of mean CTL. A two-way analysis of variance across all the subunits of Complex I showed a significant difference between CTL and sPD samples (p = 0.0003). Individual comparison of each Complex I subunit for CTL versus sPD showed a significant difference in the 30 kDa subunit (p = 0.043), 15 kDa subunit (p = 0.042), and 8 kDa subunit (p = 0.026) using the Mann-Whitney Test. Figure 1 shows that the 8 kDa and 15 kDa subunits in sPD were reduced by 80% of the corresponding CTL subunit values, with the 39 kDa subunit showing the least reduction (~30%) in sPD in comparison to CTL.

### ETC Complex II-V Subunits Are Significantly Reduced in sPD Frontal Cortex

**Figure 2:**
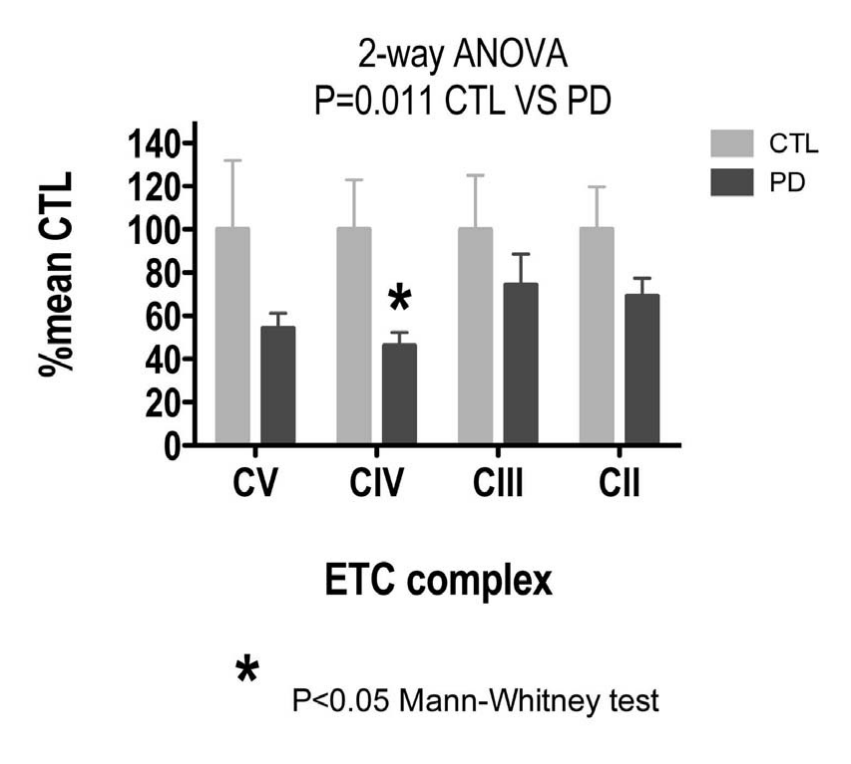
**Levels of representative subunit proteins from Complexes II-V in sPD brain mitochondria**. Individual protein band intensities were normalized to porin intensity in each sample and expressed in terms of mean CTL levels. A 2-way ANOVA across subunits and disease showed a significant interaction. Mann-Whitney test showed significant reductions for Complex IV subunit in sPD samples.

Figure 2 shows the results of Western blot assays for ETC subunits of Complexes II-V in sPD and CTL brain mitochondria, normalized to porin and expressed as percentage of mean CTL. A two-way analysis of variance across Complexes II - V showed a significant difference between CTL and sPD samples (p = 0.011). Individual comparison of CTL versus sPD showed a significant difference in Complex IV (p = 0.027) using the Mann-Whitney Test. Figure 2 shows a ~50% reduction in Complex IV and Complex V for sPD in comparison to CTLs. Complex II and Complex III show ~20% reduction in sPD versus CTLs.

### Levels of sPD Complex II, III and V Protein Subunits are Inversely Related to NADH-driven Electron Flux Rate

**Figure 3:**
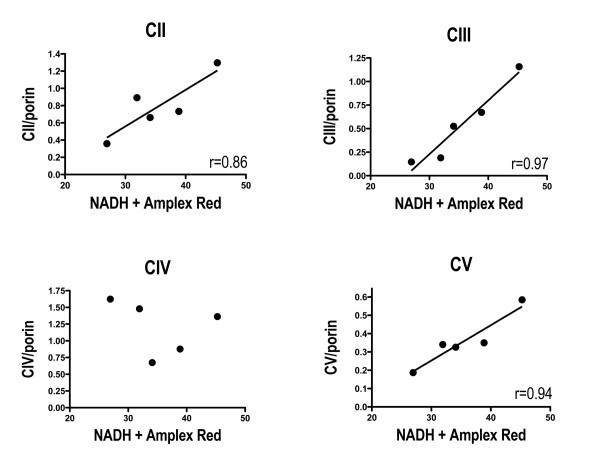
**Correlations among levels of Complexes II, III and V and NADH-driven electron flux rates in sPD brain mitochondria**. NADH-driven electron flux rates for mitochondria isolated from frontal cortex of the same cases were taken from data described in Keeney, et al. Porin-normalized levels of respiratory proteins were determined as described in **Methods**.

In our previous work we made mitochondrial preparations from many of the same sPD postmortem cortex samples used in the present study and incubated them with NADH to initiate electron flow through complex I [20]. We used a SOD1-peroxidase-Amplex Red system to detect electrons that left the ETC and reduced oxygen to superoxide. We found that these rates of NADH-driven electron flux were reduced in sPD brain mitochondria and were inversely related to amounts of oxidatively damaged complex I protein subunits. Figure 3 shows that these rates of NADH-driven electron flow were very highly correlated with levels of protein subunits from complexes II, III and V that are coded for by nuclear genes, but not from complex IV that is coded for by mtDNA. There were no correlations among NADH-driven electron flow rates and levels of any complex I protein subunits (Additional File 4).

### mtDNA Heteroplasmy Analysis Fails to Distinguish sPD from CTL mtDNA's

We used a Surveyor^® ^mismatch-cleaving nuclease approach to estimate relative burdens of low abundance heteroplasmic mutations in sPD and CTL brain mtDNA samples [23,24]. The principle behind this approach is that after PCR amplification, denatured single strands with low-abundance heteroplasmic mutations are statistically more likely to hybridize with strands containing the w.t. sequences. The resulting base mismatches are cleaved with Surveyor^® ^nuclease, yielding products whose size indicates the mutation's distance from one or the other primer sites of the PCR product amplified. This approach provides an efficient "survey" of heteroplasmic mutation numbers, abundances and sizes but only estimates location and does not define specific base alterations of each mutation.

Since all of our PCR products were ~2 kbases in size, we report results from Surveyor-cleaved products of 1000-1900 bp. In this manner we hoped both to minimize inclusion of bands that could represent the same mutation and heteroplasmies in the ~50-100 bp primer overlaps. By varying the proportion of heteroplasmy in plasmid sequences provided by the manufacturer, we found that our system could reliably detect heteroplasmic mutation abundances at the 2% level (Additional File 5). The approximate location of primers A-H on the mitochondrial genome are shown in Additional File 6.

**Figure 4:**
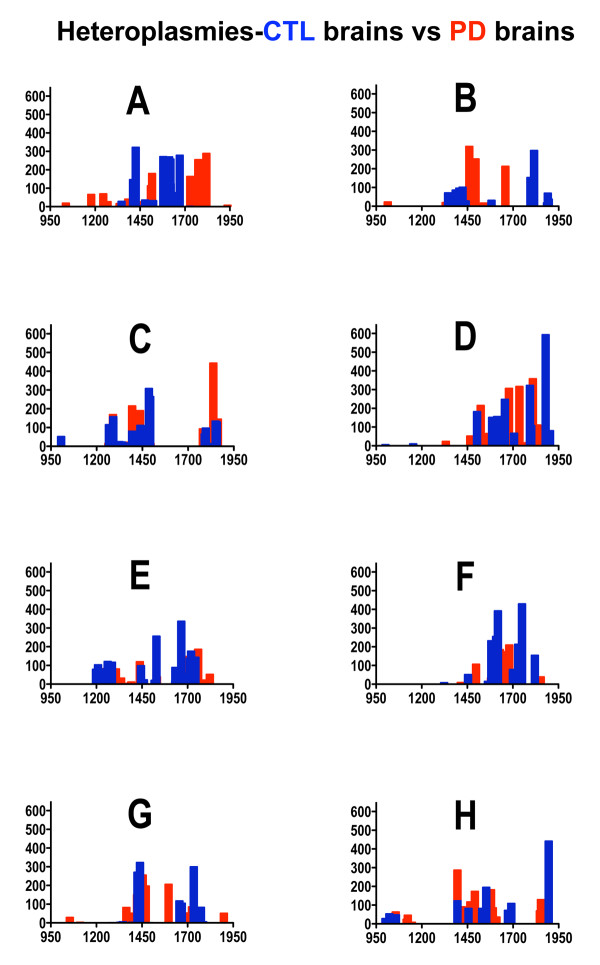
**Distributions of heteroplasmic mutations in sPD and CTL brain mtDNA**. mtDNA's from six sPD and six CTL brains were amplified using primer pairs A-H. The amplicons were subject to Surveyor Nuclease treatment and separation/analysis of products using Experion 12K DNA chips. Bands from 1000-1900 bp size are included in the Figure. The Y-axes are DNA levels and X-axes are product sizes in bp.

With this technique we found in their mtDNA's covered by the A-H primers an average of 19.0 heteroplasmies/brain in six sPD brains and 19.5 heteroplasmies/brain in six CTL brains. Figure 4 shows the distribution across primers of detected heteroplasmies and their levels from our sPD and CTL brain mtDNA's analyzed using the Surveyor^® ^nuclease approach. Two-way, non-parametric ANOVA across the variations of heteroplasmy levels and disease (sPD, CTL) for primers A-H did not reveal any significance for either variation (levels: F = 0.62, p = 0.74; disease: F = 1.90, p = 0.17) or their interaction (F = 1.10, p = 0.37).

### 8-oxoG Distribution Increases with Age in Human sPD Postmortem Brain

We estimated in mtDNA from our brain samples the distribution of 8-oxoG residues, a most commonly formed oxidized nucleoside derivative that is increased in mtDNA of vulnerable PD nigral neurons [25]. We utilized an approach whereby DNA isolated from brain mitochondria was treated with human oxyguanosine glycosylase-1 (hOGG1), a component of the base-excision repair pathway that removes oxidized guanosines and creates single-strand breaks. After single-strand breaks are created, DNA polymerase will stall and PCR amplification will be inhibited.

**Figure 5:**
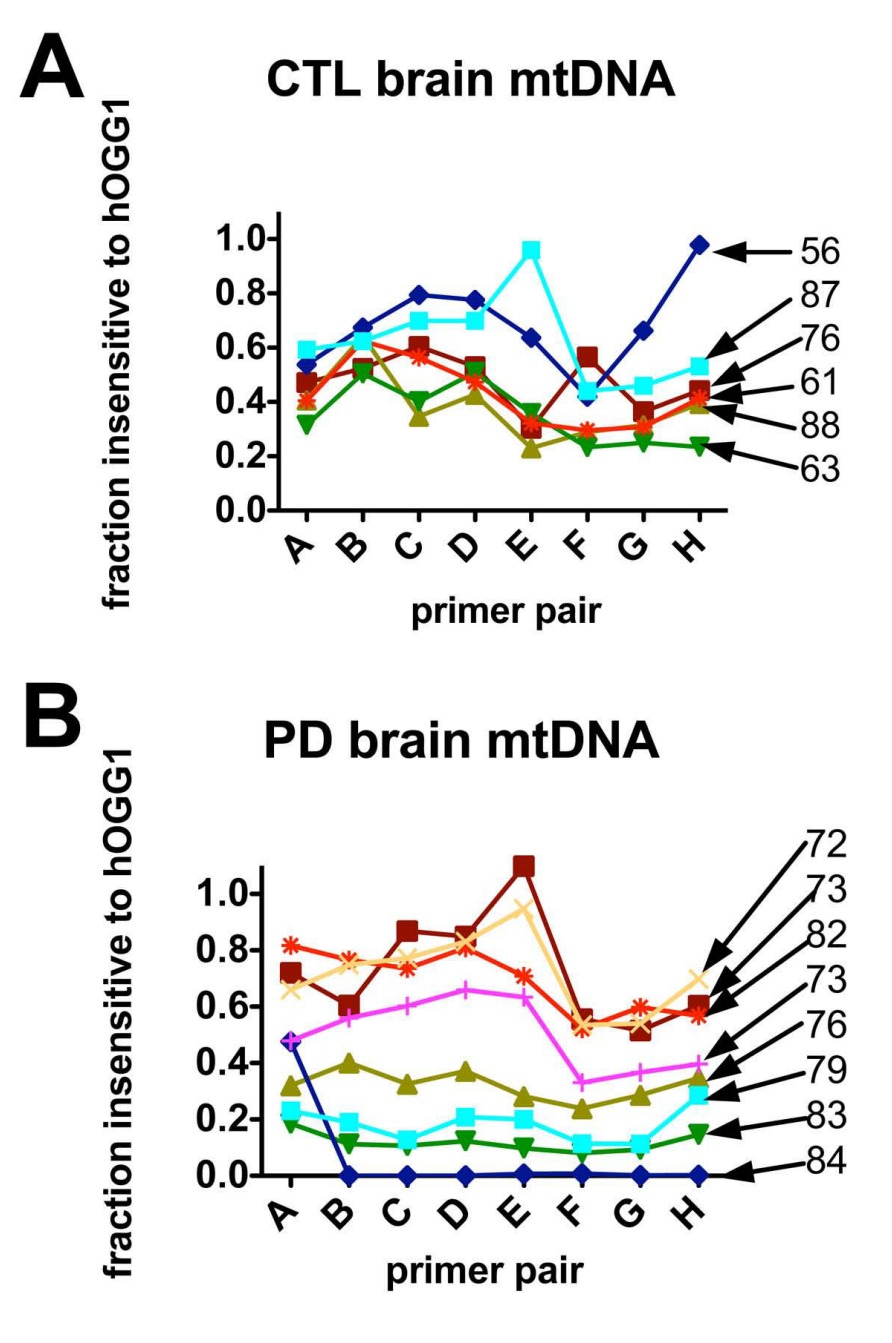
**Distribution across mtDNA of inhibition of PCR amplification from treatment with hOGG1 as a function of donor age**. mtDNA's from CTL and sPD brains were treated with hOGG1 and amplified with qPCR and primer pairs A-H. Shown are the proportional amplification for each sample and each primer pair. sPD samples showed (with one exception) an age-related in increase of hOGG1 sensitivity not seen in CTL samples. Donor ages for each sample are shown on the right.

Figure 5 shows the proportion of mtDNA retained after hOGG1 digestion following amplification by primer pairs A-H for each CTL and sPD sample. This value was obtained by dividing the qPCR calculated starting quantity (SQ) of sample treated with hOGG1 by the SQ of the same sample undigested by hOGG1. CTL samples showed mtDNA insensitive to hOGG1 from 20-80%, with greatest density of overlap at 30-60% of the genome retained. Amount of oxidative damage did not correspond to age at death in CTLs (Figure 5A) that was observed with the PD samples (Figure 5B). The sPD samples showed a wider range of relative amount of 8-oxoG oxidation from 0-90% of the mtDNA insensitive to hOGG1. Most interestingly, the level of 8-oxoG oxidation corresponded to increased age at death, with one exception. The eldest patient (age 84), exhibited almost complete oxidation of the mitochondrial genome across primers A-H, while the youngest patient (age 72) showed the least amount of oxidation in the sPD group. We did not find any correlation between apparent 8-oxoG distribution across mitochondrial genomes and levels of mtDNA heteroplasmies in either CTL or sPD brain samples (not shown).

### mtDNA copy numbers and PGC-1α expression are not altered in sPD brains

**Figure 6:**
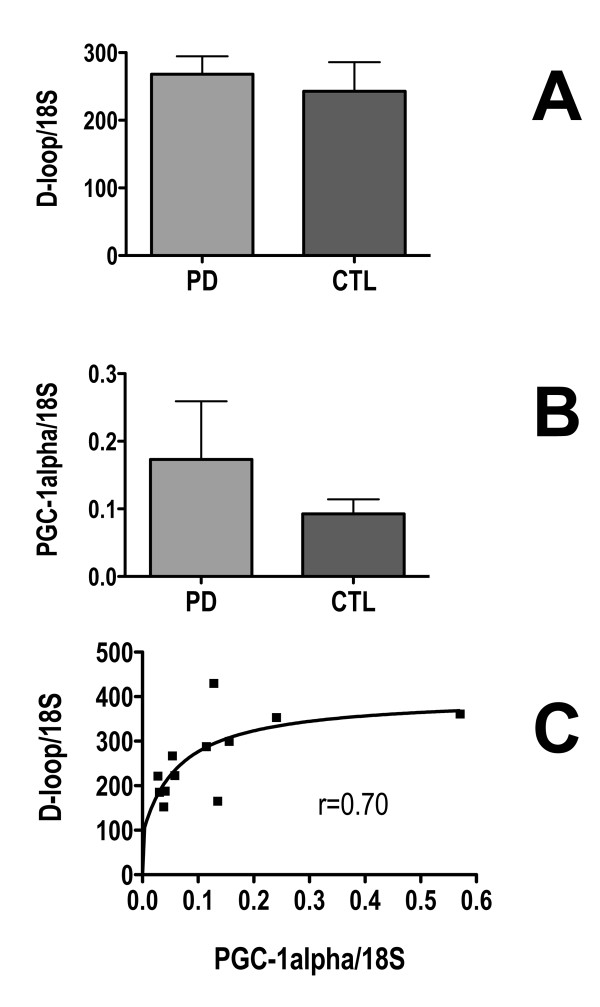
**Levels of mtDNA and PGC-1α expression in sPD and CTL brain homogenates**. mtDNA D-loop was assayed with qPCR in genomic DNA's and PGC-1α expression was assayed with qPCR in cDNA's derived from sPD and CTL brain total homogenates and normalized to 18S rRNA levels. Their relationship was best described by a two-variable rectangular hyperbola shown in C.

To search for evidence of alterations in mitochondrial biogenesis we assayed with qPCR levels of mtDNA copy number in genomic DNA and PGC-1α expression in cDNA from sPD and CTL brain homogenates (Figure 6). We did not detect any differences, and there was a non-significant trend for PGC-1α expression to be increased in sPD (Figure 6B). PGC-1α expression and mtDNA levels exhibited a rectangular hyperbolic relationship to each other (Figure 6C).

## Discussion

A fundamental limitation of postmortem brain studies in a neurodegenerative disease such as sPD is that the primary tissues analyzed result from "uncontrolled experiments". Individuals develop sPD at different ages for unclear reasons, are treated with variable doses of symptomatic drugs for varying time intervals, have different additional environmental exposures and lifestyles, possess individualized genetic backgrounds and die across a spectrum of ages from processes that may or may not be physiologically related to their sPD. What joins these specimens together in a study such as this one is the appearance of a similar clinical phenotype and the presence of common neuropathological abnormalities such as nigral cell death and Lewy body aggregates. However, that those similar clinical and pathological phenotypic characteristics exist does not require common etiologies either across individuals or disease stages. The recent discoveries of multiple monogenic parkinsonisms that share phenotypic properties with sPD and derive from mutations in seemingly disparate genes provide excellent examples of how similar neurodegenerative phenotypes can be causally distinctive. There is no reason to assume that the causes of sPD will be any less diverse.

In addition, most postmortem sPD brain samples are from persons who died in relatively advanced disease stages. Abnormalities uncovered in these specimens cannot be assumed to represent "genesis" events in disease origin. Further, sPD is an anatomically progressive disease across the entire neuraxis that is beginning to involve limbic and frontal cortex at the time nigral degeneration has progressed to yield the earliest clinical symptoms [2,4,5,7]. Abnormalities uncovered in these more rostral brain regions may or may not reflect molecular events related to nigral neurodegeneration, but we and others feel that loss of these dopaminergic neurons should longer be the sole focus of sPD investigations [26,27].

In this context, and considering these caveats, our results provide additional support to a view of sPD that considers mitochondrial dysfunction to be a substantial component of pathological progression [10,12,13,15,28]. Based on findings presented here, we propose three concepts: 1. sPD brain mitochondria eventually experience substantial misassembly of the respirasome, the proposed macro-assembly of complexes I-V that forms the functional unit of electron transport and ATP synthesis [21,22]. We did not find evidence of reduced mitochondrial biogenesis signaling as an etiology of decreased respirasome assembly; 2. sPD brain mtDNA undergoes an age-related increase in distribution of oxidative damage. This mtDNA damage is not necessary for the sPD phenotype, but it may contribute to appearance of age-related deficits in sPD such as cognitive impairment; 3. The Surveyor nuclease approach does not provide any evidence for low abundance heteroplasmic mtDNA mutations in brain as being associated with the sPD phenotype.

In our analysis of brain mitochondria isolated from PD frontal cortex, the normalized abundances of multiple subunits of Complex I examined were significantly reduced in comparison to CTLs. By normalizing to a mitochondrial mass marker (porin), we demonstrated that the 8 kDa, 15 kDa, and 30 kDa subunits were significantly decreased in sPD brain mitochondria. In our earlier work, only the 8 kDa subunit was significantly reduced, but no normalization to mitochondrial mass was utilized. In the present study we normalized each individual sample's ETC protein levels to the porin signal in that specific sample. In this manner, the relative ETC protein levels could be analyzed as a function of an abundant marker for mitochondrial mass.

The deficiency of the nuclear encoded 30 kDa subunit of Complex I, known as NADH deydrogenase (ubiquinone) iron-sulfur protein 3 (NDUFS3), is especially intriguing since this subunit is known to be catalytically active in the iron sulfur moiety of Complex I and critically involved in Complex I assembly [29,30]. The 15 kDa subunit known as NADH dehydrogenase (ubiquinone) 1 beta subcomplex subunit 4 (NDUFB4) is part of the hydrophobic protein fraction of Complex I and is notable for being one of the Complex I subunits post-translationally modified by peroxynitrite [31]. In cells from patients with a variety of Complex I deficiencies, reductions in levels of 8 kDa and 15 kDa Complex I subunits correlated better with reduced catalytic activity than did the levels of other Complex I subunits [32]. Overall our results confirm and extend earlier findings that complex I is misassembled, deficient, and dysfunctional in sPD brain mitochondria [20].

Our quantitative immunoblot study of representative members of the respirasome from mitochondria isolated from the same frontal cortex samples indicated a more widespread reduction of complexes II-V. When each complex was analyzed individually versus CTLs, the cytochrome c oxidase subunit 2 of Complex IV was significantly reduced (p = 0.027). This subunit is encoded by mtDNA and is one of three catalytic units composing the inner core of Complex IV.

The widespread reduction of multiple members of complexes I-V in sPD frontal cortex, combined with the highly correlated relationships among levels of complexes II, III and V with NADH-driven electron flux rates, suggest that the entire respirasome is deficient. Respirasomes are viewed as macromolecular assemblies of individual ETC complexes, that themselves are assemblies of multiple ETC protein subunits [21,22]. The details of respirasome assembly remain to be established, but the evolving picture is that defects in individual ETC protein subunits can disrupt assembly of that subunit's complex, which in turn interferes with normal respirasome assembly [22]. It is conceivable that the substantial deficiencies we found in Complex I subunits could be responsible for more extensive respirasome assembly defects. However, until more is known about the processes that facilitate and hinder respirasome assembly, our proposal remains speculative.

We admit that our assessment is based on indirect measurements of respirasome function, since coupled respiration is absent in frozen postmortem mitochondria. If indeed present, dysfunction of the respirasome may lead to inefficient formation of the proton gradient across the inner mitochondrial membrane needed by ATP synthase (Complex V) to create ATP, the cell's major metabolic fuel.

Our analysis of 8-oxoG distribution within the mitochondrial genome in sPD frontal cortex showed that the older cases had extensive 8-oxoG oxidation in each ~2 kbase amplicon from primers A-H (Figure 3). It is known that the presence of 8-oxoG in DNA can lead to a G → T transversions in DNA replication. Even more relevant, Brégeon et al. showed that non-dividing E. coli DNA containing 8-oxoG could cause transcriptional mutagenesis [33,34]. By cDNA sequencing obtained from mRNA transcripts, this group found that RNA polymerase incorrectly incorporated adenine across from 8-oxoG lesions [33]. They also discovered that 8-oxoG lesions could cause RNA polymerase slippage resulting in a single mRNA base deletion leading to frameshift mutations of the transcript [33]. It is theorized that these incorrectly transcribed mRNAs could lead to misfolded, dysfunctional, and reduced abundance of functional protein within these non-dividing cells. This theory potentially links our study's observation that 8-oxoG content in sPD mtDNA could be correlated to our observed reduction of the ETC respirasome protein subunits in PD frontal cortex. However, our study did not quantify the extent of 8-oxoG damage, but rather demonstrated increased distribution of 8-oxoG within the majority of the coding regions of mtDNA.

In human cell DNA replication 8oxoG:A mismatches are repaired more slowly than 8oxoG-C, resulting in increased mutagenicity from G-T transversions [35,36]. This mutagenicity of 8-oxoG may derive the ability of 8oxoG:A pairs to "fool" proofreading mechanisms of DNA polymerase [37]. This mismatch phenomenon brings in to question the fidelity of DNA sequencing methods that use prokaryotic polymerases such as the dideoxy DNA sequencing method. This method is often used when performing mtDNA heteroplasmy analyses. Because any guanine residue within the mitochondrial genome can be oxidized to 8-oxoG and thus base pair with both cytosine and adenine, some heteroplasmy analyses not adjusting for this phenomenon might show a greater mtDNA heteroplasmy than actually exists. Further studies would help to elucidate whether this miscoding phenomenon significantly has affected mtDNA heteroplasmy analyses.

Our study also showed that the prevalence of oxidatively damaged 8-oxoG residues increased with age in PD and not in CTL mtDNA. The presence of 8-oxoG damage did not act as a biomarker of sPD however, because many of the CTL samples had a considerable proportion of mtDNA containing 8-oxoG lesions. Several sPD samples also had relatively low distributions of 8-oxoG damage. Increased age and increased 8-oxoG levels in both mtDNA and nDNA have been linked in a study by Lezza et al. who examined cerebral cortex from normal human brain, and found that 8-oxoG levels were significantly increased in the cohort over the age of 70 as compared to cases below 70 years of age [38]. Alam et al. discovered a significant correlation between PD and increased levels of 8-oxoG when compared to healthy CTLs in substantia nigra [39]. Fukae et al. showed a significant increase in the percentages of nigral neurons immunoreactive to hOGG1-2a in PD when compared to neurons in substantia nigra of CTLs, progressive supranuclear palsy, and corticobasal degeneration cases [40]. This group later reported an increase in mitochondrial hMUTYH in sPD substantia nigra [41]. This enzyme removes mismatched adenines incorporated opposite 8-oxoG during replication mismatch.

Our findings complement those of Zhang et al [42]. Using immunohistochemistry for 8-oxoG, they found notable increases of cytoplasmic immunostaining in nigral neurons of sPD brains. The immunostaining was reduced by pretreatment with either DNAase or RNAase and was eliminated by pretreating with both enzymes. Their findings support the presence of increased levels of both mitochondrial DNA and RNA oxidative damage in sPD nigral neurons.

Taken together, these findings suggest a complicated interplay between mtDNA oxidation, aging, and the development of sPD; increased aging and 8-oxoG damage may be two of many factors involved in the etiology of this disease. Furthermore, the increases in hOGG1-2a in PD nigral neurons and hMUTYH in PD nigral mitochondria may demonstrate a neural compensatory mechanism when faced with increased oxidative stress damage to mtDNA.

## Conclusion

Our studies showed that sPD brain mitochondria from frontal cortex have a generalized deficiency of respirasome protein components. This finding implies a more diffuse mitochondrial bioenergetic deficit than proposals that discuss sPD as primarily possessing a deficiency at one ETC complex. We do note, however, that the greatest losses of respirasome proteins were several Complex I subunits. Thus, the functional, catalytic deficiencies found in sPD brain may be greatest at complex I, even though the larger respirasome assembly is impaired more generally at the molecular level.

Could sPD brain mtDNA be causally responsible for a more generalized respirasome deficit? Although our study does not provide any supportive evidence for this possibility, we note that our studies of sPD cybrid models have shown generalized deficiencies of multiple respirasome proteins apparently due to reduced mitochondrial biogenesis signaling [43]. Our findings in sPD cybrids suggest that mtDNA deficiency could be a contributing factor, but we note that in our current study, sPD brain mtDNA levels were not reduced, nor was there any evidence of decreased mitochondrial biogenesis signaling.

Finally, we note that we studied mitochondria purified from total brain homogenates; thus, the majority of mitochondria are likely from non-neuronal cells. It will be of value to attempt similar studies in isolated sPD brain neurons, once proteomic sensitivity has improved to allow single cell analyses.

## Methods

### Isolation of gradient purified mitochondria from human cerebral frontal cortex

Human cerebral frontal cortex samples were obtained from the Brain Resource Facility at the University of Virginia Health System. Mitochondrial isolation was performed on sPD samples (diagnosis confirmed histologically; N = 8) and age-matched controls (CTL, N = 10). Gradient purified mitochondria were obtained via a modified protocol developed by Lai and Clark [25]. 2-3 grams of frontal cortex stored at -80°C were minced in 3 mL mitochondrial isolation buffer (MIB; 0.15 M KCl, 20 mM monopotassium phosphate, 1 mM EDTA, pH 7.6) and homogenized by 60 passes with Dounce homogenizers in an additional 9 mL MIB. The homogenate was centrifuged for 3 min at 1300 × g. The supernatant was removed from the pellet and stored on ice. The pellet was resuspended in 5 mL MIB and homogenized via 30 additional passes of the Dounce homogenizers. 6 mL MIB was added to this second homogenate, which was again centrifuged at 1300 × g. This second supernatant was added to the originally isolated supernatant and centrifuged for 20 min at 17,000 × g at 4°C. The supernatant was removed and discarded. The pellet was resuspended in 30 mL MIB and centrifuged again for 20 min at 17,000 × g at 4°C. The supernatant was removed and discarded leaving a crude mitochondrial fraction.

The pellet was resuspended in 5 mL of gradient purified mitochondrial isolation buffer (GPMIB; 0.15 M KCl, 20 mM monopotassium phosphate, 1 mM EDTA, 5.5 mM 2-mercaptoethanol, 1 mM phenylmethanesulphonyl fluoride, 10% v/v of 100 × protease inhibitor cocktail from CalBioChem, pH 7.6) and homogenized with a Teflon glass homogenizer. A Ficoll solution gradient was constructed via pouring 10 mL of a 7.5% Ficoll w/w solution over 12 mL of a 10.0% Ficoll w/w solution. The crude mitochondrial fraction homogenate was poured over the Ficoll gradient and centrifuged for 37 min at 28,000 RPM at 4°C. The Ficoll solution gradient was decanted from the gradient purified mitochondrial pellet. The pellet was divided and stored at -80°C in modified radioimmunoprecipitation buffer (RIPA; 50 mM Tris-HCl pH 7.4, 1% NP 40, 0.25% sodium deoxycholate, 150 mM NaCl, 1 mM EGTA, 1 mM sodium orthovanadate, 1 mM sodium fluoride, 1 mM phenylmethanesulphonyl fluoride, 10% v/v of 100 × protease inhibitor cocktail from CalBioChem) for Western Blots and in GPMIB for subsequent DNA analyses.

### Isolation of nucleic acids and protein from gradient purified mitochondria

Gradient purified mitochondrial pellets were sonicated until completely dissolved before nucleic acid and protein isolation. Nucleic acids were isolated from gradient purified mitochondria using the AllPrep DNA/RNA Mini Kit from Qiagen using 600 μL Buffer RLT Plus. mtDNA quantification was performed using the Quant-iT dsDNA Assay Kit by Invitrogen and measured on a TECAN Genios Pro 96-well plate optical reader. For protein isolation, the mitochondrial pellets were sonicated until dissolved and vortexed every 5 min for 30 min while held on ice. The samples were centrifuged at 15,000 × g for 10 min at 4°C and supernatants containing the soluble protein fractions were collected. Protein quantification was performed using the Detergent Compatible Protein Assay Kit by Bio-Rad with BSA standards and measured using an OptiMax 96-well plate optical reader at 750 nm.

### Estimation of Heteroplasmic Mutations in mtDNA using Surveyor Nuclease

~2 kbase amplicons of the mtDNA coding region used primers A-H as described in Bannwarth, et al. [24] qPCR conditions for these amplicons used SybrGreen detection and 25 nM [primers]; cycle conditions were 95°C for 3 min, then 50 cycles of 95°C for 30 sec, 57°C for 30 sec, 72°C for 4 min. Circular mtDNA standards for qPCR analysis of amplicons from these primers were made from Roche human genomic DNA treated with Plasmid-Safe exonuclease followed by purification on Mobio columns. Roche genomic DNA served as the standard for qPCR assays of 18S rRNA gene. Human fetal brain total RNA (Clontech) was reverse transcribed into cDNA and used as a qPCR standard for estimation of relative PGC-1α levels in brain cDNA samples, which were generated with iScript reverse transcriptase (BioRad).

### Surveyor Nuclease^® ^Assay for mtDNA Mismatches

The concentration of DNA in amplicons from primers A-H was estimated after separation on a 0.8% E-gel and comparing with the Gel-Doc (BioRad) their band intensity to the intensity of the bands in the 1 kb ladder whose concentration is known. 400 ng of amplicon DNA was mixed with 2 ul of "Enhancer" and 2 ul Surveyor Nuclease^® ^to 0.2 mL PCR tubes on ice that were then mixed and incubated at 42°C for 60 min. Tubes were then transferred to ice and 2 ul of Stop Solution added. Controls (undigested amplicons) were run alongside their corresponding digests on 12K DNA chips (Experion^®^, BioRad). The Experion^® ^software calculated both bp size of DNA bands and their concentrations.

### Incubation of mtDNA with hOGG1 and quantification of 8-oxoG levels by qPCR

Incubation of mtDNA containing oxidized 8-oxoG residues with hOGG1 is predicted to produce an apurinic site at the site of oxidation due to hOGG1's N-glycosylase activity. When PCR is initiated, the Taq polymerase is unable to amplify past the 8-oxoG cleavage site where there is a single-stranded break in the DNA sugar-phosphate backbone, thus interrupting PCR amplification. Due to this phenomenon, it would be expected that undigested mtDNA would appear to have a larger starting quantity after qPCR when compared to the mtDNA from the same sample treated with hOGG1 prior to qPCR, if it is believed that the mtDNA does indeed suffer from 8-oxoG oxidation. Enzymatic incubation occurred in a 20 μL reaction volume containing 100 ng of mtDNA, 1 unit of hOGG1, 1 × Reaction Buffer (10 mM Tris-HCl, 50 mM NaCl, 10 mM MgCl2, 1 mM dithiothreitol, pH 7.9 at 25°C), and 100 μg/mL bovine serum albumin obtained from New England BioLabs. Incubation occurred at 37°C for 1 hour followed by 20 min of heat inactivation in a water bath at 90°C. All PD and CTL samples underwent incubation conditions with and without hOGG1 where matched undigested samples replaced the 1 unit of hOGG1 with a matching volume of 1 × Reaction Buffer. Quantitative PCR was performed in a 25 μL reaction volume using 5 ng of ± hOGG1 treated mtDNA sample, 0.25 μM sense/anti-sense primers, and 1 × iQ SYBR Green Supermix (2 × reaction buffer with dNTPs, iTaq DNA polymerase, 6 mM MgCl2, SYBR Green I, fluorescein, and stabalizers) from Bio-Rad. Eight primer sets developed by Bannwarth et. al labeled A-H were used and each contained ~2000 bp encircling the sense-coding portion of the mitochondrial genome (Figure 1) [26]. Quantitative PCR protocol consisted of 40 cycles with 95°C denaturation, 57°C annealing, and 72°C elongation on an iQ5 Real Time PCR Detection System by Bio-Rad, which contained software to calculate starting quantity from measured cycle threshold.

### Immunoblot for Complex I-V and Subunits of Complex I

In a 30 μL reaction volume, 25 μg of purified mitochondrial protein was added to 10 μL XT working sample buffer (XT Reducing Agent, XT Sample Buffer from Bio-Rad) and heated for 5 min at 95°C. Electrophoresis was performed using a 12-well 12% polyacrylamide Criterion XT Bis-Tris Precast Gel with 1 × XT MES Running Buffer. Precision Plus Dual Color Protein Standard containing 10 MW markers (10 kDa - 250 kDa) from Bio-Rad was used to monitor the progression of the sample down the electrophoretic gel. The iBlot Dry Blotting System from Invitrogen was used in the Western Blotting transfer of protein from the polyacrylamide gel to nitrocellulose. The nitrocellulose was washed for 3 min with phosphate buffered saline (PBS) and incubated at room temperature for 1 hour in Li-Cor Blocking Buffer. The blot was then placed in the primary antibody solution for 1 hour at room temperature. The primary antibody solution for the subunits of Complex I was made of 20 μL Li-Cor Blocking Buffer and contained 0.1% Tween 20 (polyoxyethylenesorbitan monolaurate from Bio-Rad), 0.5 μg/mL rabbit anti-SOD2 antibody (Abcam), and the following mouse monoclonal antibodies from MitoSciences: 1 μg/mL Complex I subunit 8 kDa, 0.5 μg/mL Complex I subunit NDUFB4 (15 kDa), 0.5 μg/mL Complex I subunit NDUFB8 (20 kDa), 0.5 μg/mL Complex I subunit NDUFS3 (30 kDa), 1.125 μg/mL Complex I subunit NDUFA9 (39 kDa). The primary antibody solution for Complex I-V was made of 20 μL Li-Cor Blocking Buffer and contained 0.1% Tween 20 (Bio-Rad), 0.5 μg/mL rabbit anti-SOD2 antibody (Abcam), and the following mouse monoclonal antibodies from MitoSciences: 0.5 μg/mL Complex I subunit NDUFB8 and 1:570 dilution of the MitoProfile Total OXPHOS Human WB Antibody Cocktail containing Complex I subunit NDUFB8, Complex II-FeS subunit 30 kDa, Complex III subunit Core 2, Complex IV subunit II, ATP synthase subunit alpha. After 1 hour exposure to the primary antibody solution the blot was washed 4 times for 5 min each in PBS with 0.1% Tween 20 (T-PBS). Next, the nitrocellulose was incubated for 30 min at room temperature in 20 mL of secondary antibody solution made of Li-Cor Blocking Buffer which contained 0.1% Tween 20, 33 μL 12% SDS, 1:7500 dilution of IRDye CW 800 goat anti-mouse antibody (Li-Cor) and 1:4200 dilution of IRDye CW 680 goat anti-rabbit antibody (Li-Cor). Blots were washed 4 times for 5 min with T-PBS. The Western Blots were repeated using the same protocol, but with a primary antibody solution containing 1 μg/mL mouse anti-Porin antibody (MitoSciences) and 0.5 μg/mL rabbit anti-SOD2 antibody (Abcam). Western blots were visualized using a Li-Cor Odyssey Infrared Imaging System, which contained software to quantify protein amounts based on calculated integrated intensities.

### Amplex Red Assay for NADH-driven ROS Production in Postmortem Brain Mitochondria

This assay was described in our earlier publication [20]. It is based on using NADH to drive electrons through mitochondrial complex I and into the ETC. Electrons leaving the ETC react with ambient oxygen to form superoxide, which is then dismutated to hydrogen peroxide by both endogenous mitochondrial SOD2 and added SOD1. The hydrogen peroxide formed, with added catalase, oxidizes Amplex Red dye to a fluorescent derivative. The rate of oxidized Amplex Red product formed over time is assayed in a plate reader at 37 degrees.

### Statistical Analyses

Statistical analyses were performed using InStat software from GraphPad and JMP8 software from SAS Institute Inc.

## List of Abbreviations

8-oxoG: 8-oxyguanine; CTL: control; hOGG1: human oxyguanine glycosylase-1; mtDNA: mitochondrial DNA; sPD: sporadically occurring Parkinson's disease.

## Competing interests

The authors declare that they have no competing interests.

## Authors' contributions

CRA and JPB designed experiments; CRA, SKM, LDD, PMK and JPB carried out experiments and analyzed data; CRA and JPB wrote the manuscript. All authors read and approved the manuscript.

## Supplementary Material

Additional file 1 **Clinical characteristics of control cases**. Clinical and demographic characteristics of control cases. Click here for file

Additional file 2 **Clinical characteristics of Parkinson's disease cases**. Clinical and demographic characteristics of Parkinson's disease cases. Click here for file

Additional file 3 **Comparison of control and Parkinson's disease cases**. statistical comparison of control and Parkinson's disease cases for age, gender and post-mortem interval. Click here for file

Additional file 4 **Lack of relationship among levels of Complex I subunits and NADH-driven electron flux rates in sPD samples**. Shown are data from the same sPD samples used for Figure 3. Click here for file

Additional file 5 **Sensitivity to detect low abundance heteroplasmy with Surveyor Nuclease approach**. Sensitivity to detect low abundance heteroplasmy with Surveyor Nuclease approach. Shown are data from manufacturer supplied plasmids mixed in varying proportions and analyzed. (data courtesy of Caitlin Quigley). Click here for file

Additional file 6 **Cartoon showing approximate locations of amplicons for primers A-H**. Cartoon showing approximate locations of amplicons for primers A-H. Click here for file
