# Supplementary material for: Parkinson's disease brain mitochondria have impaired respirasome assembly, age-related increases in distribution of oxidative damage to mtDNA and no differences in heteroplasmic mtDNA mutation abundance
Source: Mol Neurodegener. 2009 Sep 23;4:37. doi: 10.1186/1750-1326-4-37 (PMC2761382; doi:10.1186/1750-1326-4-37)
Supplement: Additional file 1 — Clinical characteristics of control cases. Clinical and demographic characteristics of control cases. [file 1750-1326-4-37-S1.doc]

| **Supplemental Table 1: Clinical Characteristics of Control Cases** | | | | |
| --- | --- | --- | --- | --- |
| **Case** | **Sex** | **Age** | **PMI (hours)** | **Medical History** |
|  |  |  |  |  |
| 122 | M | 76 | 12 | aortic stenosis; death from cardiogenic shock/renal failure |
| 125 | M | 48 | 2.5 | MI, unstable angina; death from cardiac arrest 3 days s/p 3 vessel CABG |
| 137 | M | 88 | 5.5 | stroke, MI, CAD, COPD; death from pneumonia |
| 144 | M | 63 | 12 | aortic aneurysm, HTN, COPD, hepatic atrophy; death from ruptured aneurysm |
| 145 | M | 59 | 2.5 | squamous cell carcinoma of larynx with mets; death from respiratory failure |
| 147 | M | 71 | 5 | adenocarcinoma of the prostate w/bony mets; death from cachexia/tumor burden |
| 164 | F | 53 | 14 | cardiomyopathy; death from cardiac arrest |
| 213 | M | 61 | 6 | idiopathic pulmonary fibrosis, HTN, HL, GERD; death from pneumonia |
| 216 | F | 87 | 13 | HTN, osteoporosis; death from acute MI |
| 228 | F | 64 | 14 | uterine mullerian tumor w/mets to lungs; death from pneumonia and resp. failure |
| **Table 1.** PMI, postmortem interval; M, male; F, female; MI, myocardial infarction; | | | | |
| CABG, coronary artery bypass graft; CAD, coronary artery disease; COPD, chronic obstructive pulmonary disease; | | | | |
| HTN, hypertension; mets, metastases; HL, hyperlipidemia; GERD, gastroesophageal reflux disease | | | | |
