# Supplementary material for: Parkinson's disease brain mitochondria have impaired respirasome assembly, age-related increases in distribution of oxidative damage to mtDNA and no differences in heteroplasmic mtDNA mutation abundance
Source: Mol Neurodegener. 2009 Sep 23;4:37. doi: 10.1186/1750-1326-4-37 (PMC2761382; doi:10.1186/1750-1326-4-37)
Supplement: Additional file 2 — Clinical characteristics of Parkinson's disease cases. Clinical and demographic characteristics of Parkinson's disease cases. [file 1750-1326-4-37-S2.docx]

| **Supplemental Table 2: Clinical Characteristics of Parkinson's Disease Cases** | | | | |
| --- | --- | --- | --- | --- |
| **Case** | **Sex** | **Age** | **PMI (hours)** | **Medical History** |
|  |  |  |  |  |
| 168 | F | 73 | 15.5 | 23 year history of PD, depression; cortical plaques did not meet criteria for AD |
| 170 | M | 76 | 12.75 | orthostatic hypotension; no evidence of MSA at autopsy |
| 171 | M | 83 | 5.5 | 4 year history of PD, hypothyroidism; no evidence of AD or MSA at autopsy |
| 172 | M | 84 | 10 | 12 year history of PD, depression; no evidence of AD or MSA at autopsy |
| 174 | F | 82 | 13.5 | depression; discoid lupus erythematosus |
| 175 | M | 79 | 16.5 | 10 year history of PD, possible CLL, neurogenic bladder; no AD at autopsy |
| 187 | F | 73 | 9.5 | >20 year history of PD |
| 203 | M | 72 | 18 | 26 year history of PD, s/p CABG×5, history of smoking |
| **Table 2.** PMI, postmortem interval; M, male; F, female; PD, Parkinson's disease; AD, Alzheimer's disease; | | | | |
| MSA, multisystem atrophy; CLL, chronic lymphocytic leukemia; CABG, coronary artery bypass graft | | | | |
|  |  |  |  |  |
