# Supplementary material for: Parkinson's disease brain mitochondria have impaired respirasome assembly, age-related increases in distribution of oxidative damage to mtDNA and no differences in heteroplasmic mtDNA mutation abundance
Source: Mol Neurodegener. 2009 Sep 23;4:37. doi: 10.1186/1750-1326-4-37 (PMC2761382; doi:10.1186/1750-1326-4-37)
Supplement: Additional file 3 — Comparison of control and Parkinson's disease cases. statistical comparison of control and Parkinson's disease cases for age, gender and post-mortem interval. [file 1750-1326-4-37-S3.doc]

| **Supplemental Table 3: Comparison of Parkinson's Disease and Control Cases** | | | | | | | |
| --- | --- | --- | --- | --- | --- | --- | --- |
| **Characteristic** | **PDs (N=8)** | | **CTL (N=10)** | | **p Value** | | **Test Used** |
|  |  | |  | |  | |  |
| Age - years | 77.8 ± 4.9 | | 67.0 ± 13.4 | | 0.0753 | | Wilcoxon rank sum test |
| Male Sex - no. (%) | 5 (0.625) | | 7 (0.700) | | 0.737 | | Pearson chi squared test |
| PMI - hours | 12.66 ± 4.15 | | 8.65 ± 4.77 | | 0.109 | | Wilcoxon rank sum test |
| **Table 3.** Statistical analyses of Parkinson's Disease versus Control cases characteristics. | | | | | | | |
|  |  |  | |  | |  | |
|  |  |  | |  | |  | |
