# Supplementary figures and images for: Parkinson's disease brain mitochondria have impaired respirasome assembly, age-related increases in distribution of oxidative damage to mtDNA and no differences in heteroplasmic mtDNA mutation abundance
Source: Mol Neurodegener. 2009 Sep 23;4:37. doi: 10.1186/1750-1326-4-37 (PMC2761382; doi:10.1186/1750-1326-4-37)

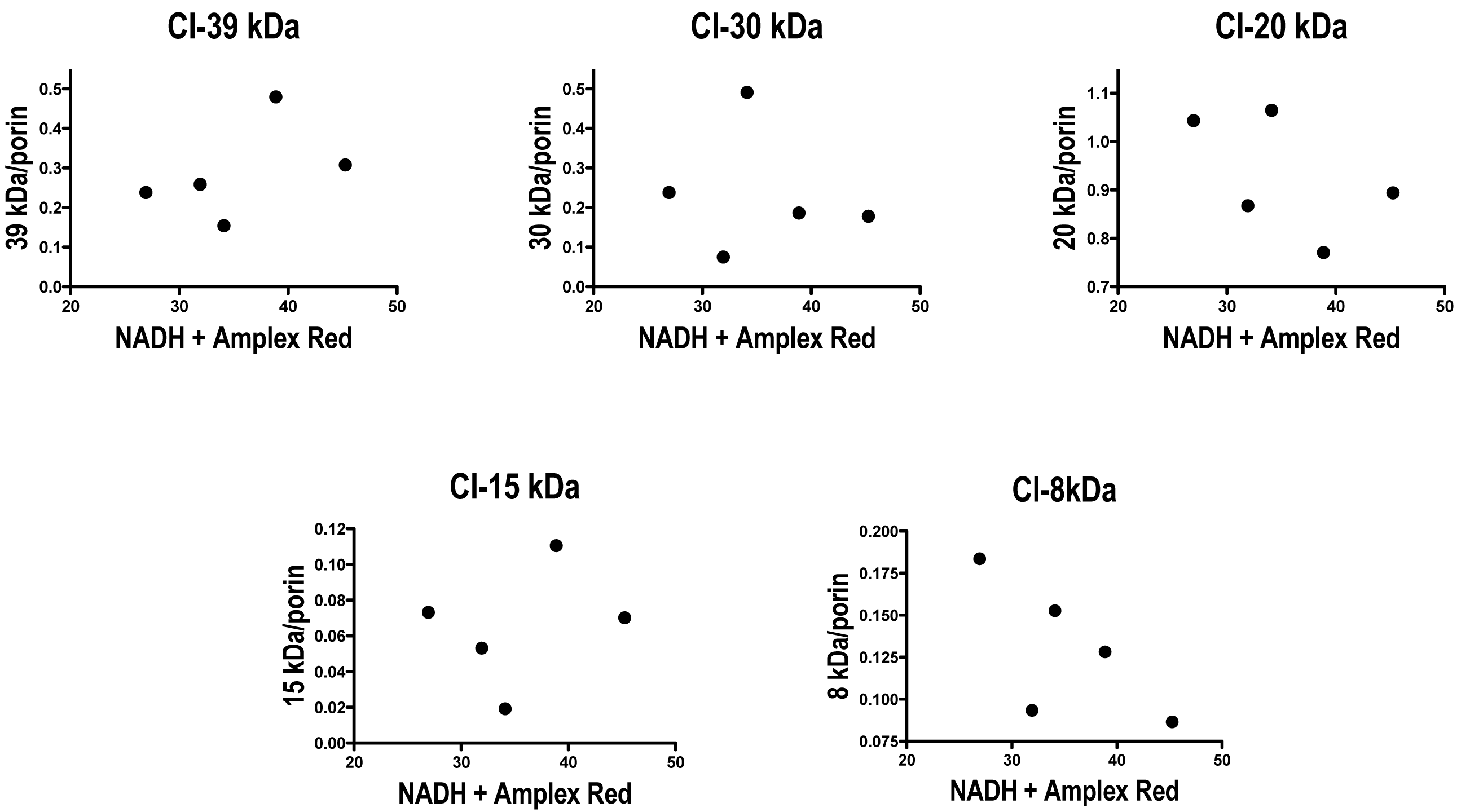

Supplement: Additional file 4 — Lack of relationship among levels of Complex I subunits and NADH-driven electron flux rates in sPD samples. Shown are data from the same sPD samples used for Figure 3. [file 1750-1326-4-37-S4.tiff]

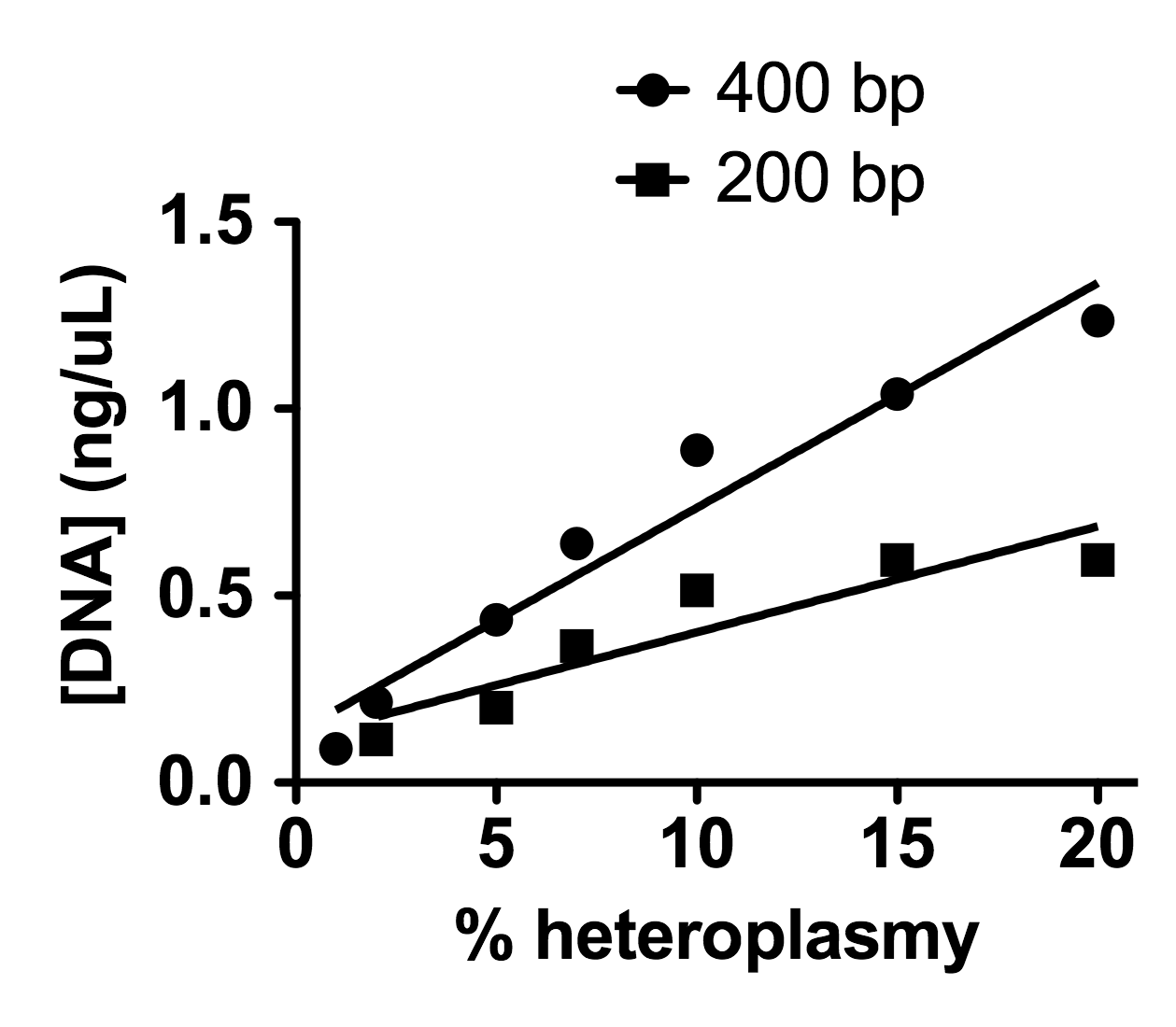

Supplement: Additional file 5 — Sensitivity to detect low abundance heteroplasmy with Surveyor Nuclease approach. Sensitivity to detect low abundance heteroplasmy with Surveyor Nuclease approach. Shown are data from manufacturer supplied plasmids mixed in varying proportions and analyzed. (data courtesy of Caitlin Quigley). [file 1750-1326-4-37-S5.tiff]

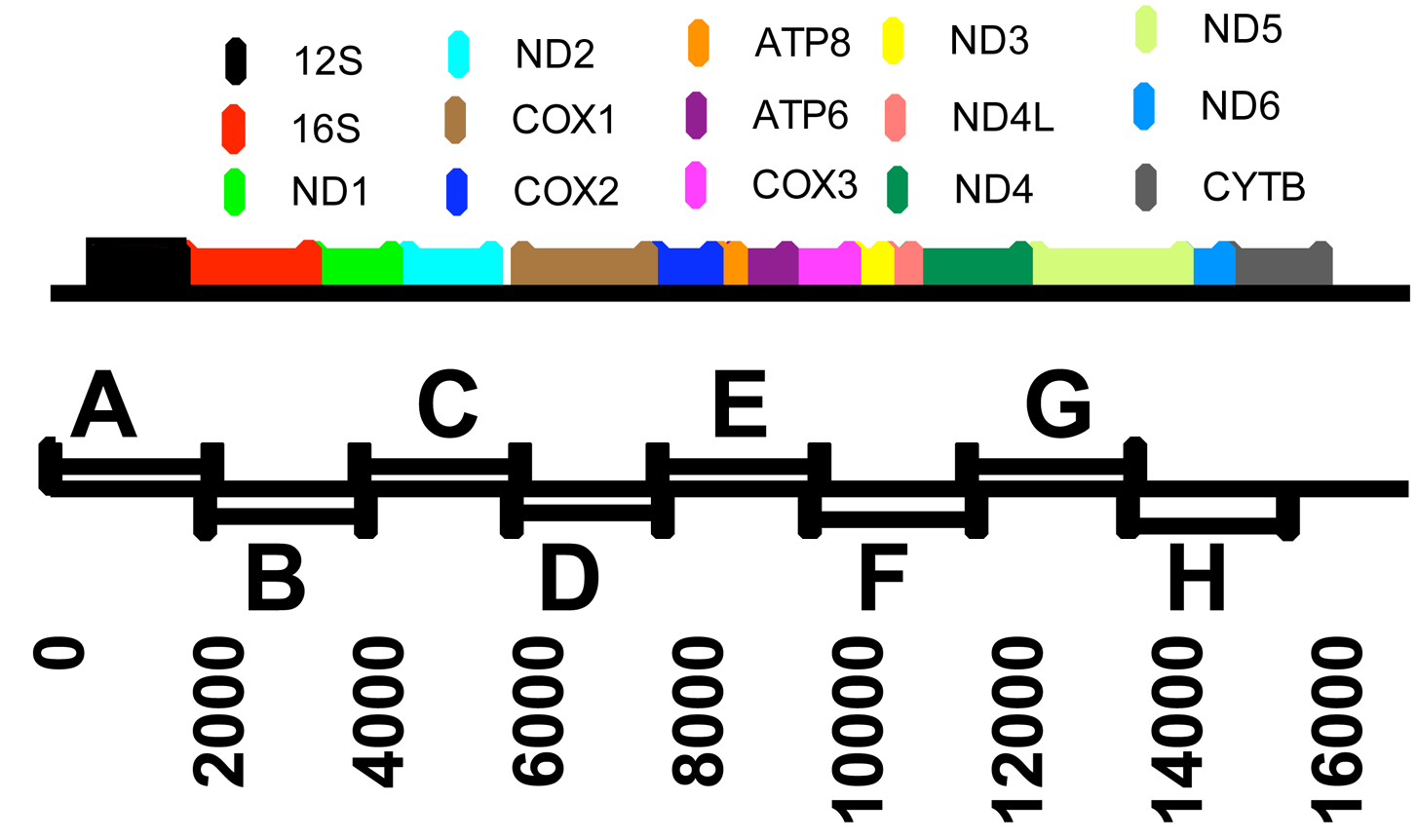

Supplement: Additional file 6 — Cartoon showing approximate locations of amplicons for primers A-H. Cartoon showing approximate locations of amplicons for primers A-H. [file 1750-1326-4-37-S6.tiff]
